# Unveiling the role of ASPP1 in cancer progression: pan-cancer bioinformatics and experimental validation in colorectal cancer

**DOI:** 10.3389/fonc.2024.1529809

**Published:** 2025-01-02

**Authors:** Keyuan Xiao, Xiang Li, Ihsan Ullah, Wenqing Hu, Kaiqiang Wang, Fan Yang, Chengyu Yang, Chunqi Feng, Liang Zong, Xinghua Li

**Affiliations:** ^1^ Clinical Research Center, Changzhi People’s Hospital Affiliated to Changzhi Medical College, Changzhi, China; ^2^ School of Pharmacy, Heilongjiang University of Chinese Medicine, Harbin, China; ^3^ School of Pharmacy, Changzhi Medical College, Changzhi, China; ^4^ College of Life Sciences, Shanxi University, Taiyuan, China; ^5^ School of Pharmacy, Shanxi Medical University, Taiyuan, China

**Keywords:** ASPP1, pan-cancer analysis, tumor suppression, immune modulation, colorectal cancer

## Abstract

**Background:**

The Apoptosis-Stimulating Protein of P53 (ASPP) family contributes to apoptosis regulation and tumor suppression, with ASPP1 influencing processes like cancer cell proliferation, invasion, and migration. Its expression varies across cancer types, suggesting a potential role in oncogenesis.

**Methods:**

This study investigates ASPP1’s role across various cancers using a comprehensive bioinformatics approach. Data were extracted from public resources, including The Cancer Genome Atlas (TCGA), GTEx, and the Human Protein Atlas, and analyzed via tools such as cBioPortal, GEPIA, and TIMER2. Statistical and network analyses were performed with R, Cytoscape, and Hiplot. ASPP1’s function in colorectal cancer was further explored through *in vitro* assays, including qRT-PCR, Western blotting, colony formation, Transwell, and wound healing.

**Results:**

ASPP1 expression exhibited significant variability across different cancer types, with marked associations with patient outcomes, particularly overall survival (OS) and disease-specific survival (DSS) across several cancer types. In-depth protein-protein interaction (PPI) analysis revealed ASPP1’s involvement in apoptosis and cancer progression networks. Functional enrichment analysis further linked ASPP1 to key apoptotic signaling pathways and transcriptional regulatory processes, underscoring its potential impact on tumor biology. Additionally, the expression of ASPP1 correlates with immune cell infiltration patterns, including cancer-associated fibroblasts and various immune markers, suggesting roles in immune response modulation. *In vitro* assays with colorectal cancer cell lines revealed significantly lower ASPP1 expression levels compared to normal colon cells (HCM460), and ASPP1 overexpression experiments showed a marked reduction in colorectal cancer cell proliferation, colony formation, invasion, and migration abilities. These cellular findings align with the bioinformatics predictions, highlighting ASPP1’s role as a suppressor of metastatic traits in colorectal cancer.

**Conclusion:**

This study highlights ASPP1 as a forecasting biomarker in the colorectal cancers and potentially across other cancers. The findings support ASPP1’s involvement in tumor biology, particularly regarding cell proliferation and metastatic potential, establishing a foundation for further investigation into its therapeutic relevance.

## 1 Introduction

Cancer continues to be a formidable health challenge worldwide, with both incidence and mortality rates remaining high despite significant advances in early detection, targeted therapy, and immunotherapy (1). Cancer is a multifaceted disease defined by the uncontrolled growth of cells, which gain the ability to invade local tissues and spread (metastasize) to distant sites in the body (2). This complexity underscores an urgent need for novel therapeutic targets and a deeper understanding of the molecular mechanisms that drive tumor initiation and progression. A promising strategy to explore these mechanisms is through pan-cancer analysis, an approach that integrates and examines multi-omics data from a broad spectrum of cancer types. By analyzing multiple cancers collectively, researchers can identify overarching molecular patterns and pathways that may be pivotal to oncogenesis and tumor evolution (3). Pan-cancer analysis has enabled the discovery of key genetic alterations and pathways that are commonly dysregulated, paving the way for identifying therapeutic targets with potentially broad applications.

One critical biological process that is often disrupted in cancer is apoptosis, or programmed cell death, which serves as a fundamental mechanism for maintaining cellular homeostasis by eliminating damaged or aberrant cells (4). Apoptosis functions as a safeguard against malignancy; however, cancer cells frequently develop mechanisms to evade apoptosis, thereby enabling their survival and unchecked proliferation (5). This evasion is often facilitated by alterations in apoptotic signaling pathways, which can be triggered by a range of cellular stressors such as DNA damage, oxidative stress, and activation of cell surface death receptors (6). Among the key regulators of apoptosis is the ASPP (Apoptosis-Stimulating Protein of p53) protein family, which includes ASPP1, ASPP2, and iASPP. These proteins are of particular interest due to their interaction with the tumor suppressor protein p53, a central figure in maintaining genomic stability through its roles in DNA repair, cell cycle control, and apoptosis induction (7–12). ASPP1 and ASPP2 enhance the pro-apoptotic function of p53, promoting cell death in response to damage, while iASPP acts as a negative regulator, inhibiting apoptosis and thus potentially contributing to tumor progression (13, 14).

Dysregulation of ASPP family proteins has been observed in various cancer types, often correlating with poor prognosis. For instance, reduced expression of ASPP1 has been associated with worse outcomes in cancers such as acute lymphoblastic leukemia, breast cancer, hepatocellular carcinoma, clear-cell renal cell carcinoma, and colorectal cancer (10, 11, 15–17). These findings suggest that ASPP proteins, especially ASPP1, may serve as valuable biomarkers for prognosis and potential therapeutic targets. Alterations in ASPP1 expression or function may provide insights into the mechanisms through which cancers develop resistance to apoptosis and continue to proliferate. Understanding these patterns could inform therapeutic strategies aimed at reactivating apoptotic pathways in tumor cells, thereby enhancing the efficacy of existing treatments.

This study aims to perform a comprehensive pan-cancer analysis of ASPP1 by examining its expression patterns and genetic alterations across multiple cancer types. By leveraging multi-omics datasets—including gene expression, DNA methylation, and mutation profiles—we seek to elucidate the role of ASPP1 in cancer development, progression, and treatment response. Given ASPP1’s involvement in modulating apoptosis and its dysregulation across several cancers, a better understanding of its function could aid in the development of targeted therapies and contribute to the advancement of precision oncology. Preliminary *in vitro* findings have shown that ASPP1 overexpression can reduce proliferation and metastatic potential in colorectal cancer cells, further suggesting its role as a tumor suppressor and therapeutic target. Through this study, we hope to provide new insights that could lead to innovative cancer therapies and personalized treatment strategies, potentially improving patient outcomes across a range of malignancies.

## 2 Materials and methods

### 2.1 Materials and reagents

The human colorectal cancer (CRC) cell lines HT-29, SW480, and HCT116, along with the normal human intestinal epithelial cell line NCM460, were cultured in MCCOY’S 5A, L-15, and RPMI 1640 media (Solarbio, China) with the addition of fetal bovine serum (CELL-BOX, China) under controlled conditions. Transfections were performed using Lipofectamine 8000 (Beyotime Biotechnology, Shanghai, China), with penicillin-streptomycin added to ensure sterility. For cell invasion experiments, we employed a 24-well Transwell system (Corning, NY, USA; 8-µm pore size) coated with Matrigel (Corning, NY, USA). mRNA quantification was performed using the Two-Step RT-qPCR Kit (Seven Biotech, Beijing, China) after total RNA was extracted using the RNA Extraction Kit (Seven Biotech, Beijing, China). The primary antibodies targeting β-actin were supplied by Beyotime Biotechnology (Shanghai, China), and the antibodies targeting ASPP1 were supplied by Affinity Biosciences (Jiangsu, China). In addition, Beyotime Biotechnology (Shanghai, China) provided HRP-conjugated secondary antibodies, including anti-rabbit IgG and anti-mouse IgG.

### 2.2 Expression analysis of ASPP1 in pan-cancer

We retrieved the human protein atlas database (https://www.proteinatlas.org/) with ASPP1(PPP1R13B) at summary and tissue groups in order to get the organs and tissues’ RNA and protein expression summary (18). There are graphs showing the expression in different organizations at RNA and protein levels. According to their functional characteristics, tissues are divided into color-coded groups. The image data pages for specific tissues are linked once you select them from this list. Based on knowledge-based annotation, data on protein expression is shown for 44 tissues. Using tissue groups, which consist of tissues with similar functional properties, color coding is carried out. In the mouse-over function, the protein scores for the analyzed cell types are displayed for the selected tissue. The image data can be accessed by clicking on the name of the tissue or the bar. Detailed descriptions of protein annotation are available in Assays & annotations. Pan-cancer datasets from TCGA and GTEx were utilized to examine ASPP1 mRNA expression in tumor and normal tissue samples. TPM data were log2-transformed for subsequent analyses, allowing comparisons between unpaired tumor and normal tissues across various cancer types. Expression charts were generated separately for each dataset to illustrate ASPP1 levels in cancerous and non-cancerous tissues. Using the TCGA database, we also assessed ASPP1 expression in paired tumor and adjacent normal tissues, excluding cancer types that lacked corresponding normal samples in TCGA. Data was analyzed with Wilcoxon signed-rank test to determine statistical differences (19).

### 2.3 Exploring ASPP1’s Receiver Operating Characteristic curve across various cancer types in cancers

This study assesses ASPP1’s diagnostic value in cancers types. ASPP1 ROC curves data were obtained and extracted from the TCGA database using the STAR workflow in FPKM format. Perform ROC analysis on data using the pROC [1.18.0] package to log2 transformation (value+1), and visualize the results using ggplot2 [3.3.6] to plot The Area under Curve (AUC) (20). It is better to have an AUC value closer to 1 as it signifies better performance from the classifier. An AUC between 0.7 and 0.9 is regarded as good accuracy, while an AUC of 1 or more is regarded as high accuracy.

### 2.4 Exploring ASPP1 expression’s impact on cancers survival

To assess the prognostic relevance of ASPP1 across 33 cancer types, Kaplan–Meier survival analysis (http://kmplot.com/) was applied. Disease-specific survival (DSS), overall survival (OS) and progression-free interval (PFI) were analyzed using Cox regression models. We visualized the total sample size, hazard ratios (HRs) with 95% confidence intervals, and p-values from the Kaplan–Meier analysis in forest plots created using ggplot2 (21, 22).

### 2.5 ASPP1 expression: impact on cancer staging, immune, and molecular subtypes

The expression of ASPP1 gene was correlated with various clinical pathological stages (stages I, II, III, IV) by using the GEPIA Pathological Stage Plot online tool (http://gepia.cancer-pku.cn/) for all cancer types. For plotting, we used log2(TPM + 1) to transform the expression data (23). We evaluated the ASPP1 expression and molecular subtypes, immune subtype by searching the TISIDB in subtypes page (http://cis.hku.hk/TISIDB/). In order to construct the Violin diagram, we selected cancer types using the Kruskal-Walli’s test score based on the immune subtypes and molecular subtypes bar charts (24).

### 2.6 Functional enrichment analysis of ASPP1

To conduct functional gene enrichment analysis, we first used STRING to identify ASPP1-binding proteins and construct its protein–protein interaction network. Next, we employed the Similar Genes Detection tool in GEPIA2 to retrieve ASPP1-correlated genes across various cancer types. Using the clusterProfiler [v4.4.4] R package, we then performed KEGG and GO enrichment analyses, visualizing results as bubble charts created with ggplot2 [v3.3.6]. We downloaded RNAseq data processed through TCGA STAR in TPM format from the TCGA database to analyze gene correlations. Correlation analyses were subsequently conducted, and results were presented as scatter plots using ggplot2 [v3.3.6] (25).

### 2.7 Genomic alterations of ASPP1 in pan-cancer

We queried the TCGA PanCancer Atlas studies for ASPP1 using cBioPortal (https://www.cbioportal.org/). The ‘Oncoprint’ module was applied to examine the frequency of ASPP1 gene alterations. Additionally, the ‘Cancer Types Summary,’ ‘Mutations,’ and ‘mRNA vs. Study’ modules were utilized to explore somatic mutations and ASPP1 genomic profiles across cancer types. Specific mutation sites were identified via the ‘Mutations’ module (18, 26).

### 2.8 Immune infiltration analysis in cancers

At TIMER2 (http://timer.comp-genomics.org/), we accessed the ‘Immune’ section, entering ASPP1 and cancer-associated fibroblasts to examine the relationship between immune cell infiltration and genomic alterations. The EPIC, MCPCOUNTER, XCELL, and TIED algorithms were used to estimate immune infiltration in TCGA samples. The resulting immune infiltration heatmap was downloaded, and scatter plots displaying purity and infiltration levels were generated by selecting Spearman’s p-values from the output table (27).

### 2.9 Immunogenomic analyses of ASPP1 in cancers

Similar to previous data acquisition methods, we obtained biochemical characteristics and RNA-sequencing results for ASPP1 from the TCGA dataset. To investigate immune-checkpoint and immune-activation genes in immune environment, we employed an R software package that integrates six advanced algorithms: MCP-counter, xCell, TIMER, CIBERSORT, quanTIseq, and EPIC. The expression levels of immune checkpoint transcripts were assessed using markers such as PDCD1, CD274, IDO1, SIGLEC15, HAVCR2, LAG3, CTLA4, and PDCD1LG2 (28). We also analyzed the correlations between ASPP1 expression and eight genes that play a role in immune checkpoints using the dataR package [4.0.3]. Data from two groups were analyzed using Wilcox’s test. P values<0.05 were considered statistically significant (*p < 0.05). Furthermore, ASPP1 and TILs, Immunoinhibitors, Immunostimulators, MHC molecules, Chemokine receptors, and Chemokines were analyzed using the TISIDB database modules Immunomodulators and Chemokines (24).

### 2.10 Experimental validation *in vitro*

#### 2.10.1 Cell culture and transfection

This study employed human colorectal cancer cell lines HT-29, SW480, HCT116, and the normal human intestinal epithelial cell line NCM460. All cell lines underwent routine mycoplasma testing to ensure a contamination-free environment. HT-29 and HCT116 cells were cultured in DMEM with 10% FBS and 1% penicillin-streptomycin at 37°C in a 5% CO_2_ incubator, while NCM460 cells were maintained in RPMI 1640 medium under identical conditions. To induce ASPP1 overexpression, the ASPP1 gene was cloned into a pEGFP-C1 vector and then transfected into HT-29 cells with Lipofectamine 8000. The cells transfected with the ASPP1-pEGFP-C1 construct formed the experimental group, while the control group consisted of cells transfected with the empty pEGFP-C1 vector.

#### 2.10.2 Quantitative Real-Time Polymerase Chain Reaction

qRT-PCR was used to analyze ASPP1 mRNA expression in both colorectal cancer and normal intestinal epithelial cells. Human colorectal cancer cell lines HT-29, SW480, HCT116, and the normal epithelial cell line NCM460 were cultured and harvested for analysis. Total RNA was extracted from the cells using the SevenFast Total RNA Extraction Kit. Following extraction, reverse transcription was carried out, and quantitative real-time PCR (qRT-PCR) was performed using the SevenFast Two-Step RT and qPCR Kit. Fluorescence detection was confirmed through melting curve analysis, and the mRNA levels of ASPP1 were quantified by the 2^–ΔΔCT method, with β-actin as the internal reference gene. The specific primer sequences for ASPP1 and β-actin used in this study as follows:

ASPP1-F: 5′-ACCCTCTCAGAGCTCCAAGATAT-3′.
ASPP1-R: 5′-CTTGTCCTCTCATTGCACGAATT-3′.
β-actin-F: 5′-CAGATGTGGATCAGCAAGCAGGA-3′.
β-actin-R: 5′-CGCAACTAAGTCATAGTCCGCCTA-3′.

#### 2.10.3 Western blotting

After two washes with PBS, total protein was extracted using RIPA buffer, and concentrations were determined with a BCA protein assay kit. Aspp1 and β-Actin proteins were separated by SDS-PAGE and transferred to PVDF membranes for overnight incubation with primary antibodies, followed by secondary antibodies conjugated to HRP. And then we quantified grayscale values using Image Lab.

#### 2.10.4 Colony formation assay

The assay was conducted with two groups: a control group and an ASPP1 overexpression group. Logarithmically growing cells were treated with 0.25% trypsin to obtain single-cell suspensions, which were then counted and resuspended in medium enriched with 10% fetal bovine serum. A total of 1000 cells per well were seeded in six-well plates and cultured for 14 days until distinct colonies were visible. Colony numbers were analyzed using ImageJ software, and differences between the two groups were statistically evaluated.

#### 2.10.5 Wound healing assay

Cell migration capability was evaluated through a wound healing assay. Control and ASPP1 overexpression groups were seeded at a density of 1.2 × 10^6 cells per well in six-well plates. Once the cells reached full confluence, a scratch was made with a 10-μL pipette tip. After washing with PBS to remove debris, the cells were incubated in serum-free medium for 48 hours. Images were captured at 0, 24, and 48 hours using a light microscope. Migration rates were analyzed using ImageJ software.

#### 2.10.6 Transwell assay

A Transwell assay was conducted to measure cell invasion. Matrigel was pre-thawed at 4°C and then applied to the upper surface of the Transwell insert with 50 µL, which was incubated for 3 hours until a visible white coating formed. Cells from the control and ASPP1-overexpressing groups were prepared at a density of 6 × 10^^4^ cells in 200 µL of serum-free medium, while medium with 10% FBS was added to the lower chamber. After 48 hours of incubation, the inserts were removed, washed with PBS, and fixed in 4% paraformaldehyde for 15 minutes. The cells were then stained with 0.1% crystal violet for 20 minutes to enhance visibility. Cell counts of those that had migrated through the membrane were performed under an inverted microscope, and ImageJ software was employed for image analysis.

#### 2.10.7 Statistical analysis

Based on three or more independent experiments, data are expressed as mean ± SD. One-way ANOVA was used to compare multiple groups, and statistical analyses were conducted using GraphPad Prism 8.0 software. Differences between groups were evaluated with single-factor ANOVA followed by Dunnett’s test, with a significance level set at α = 0.05.

## 3 Results

### 3.1 ASPP1 expression landscape and pan-cancer expression

**Figure 1:**
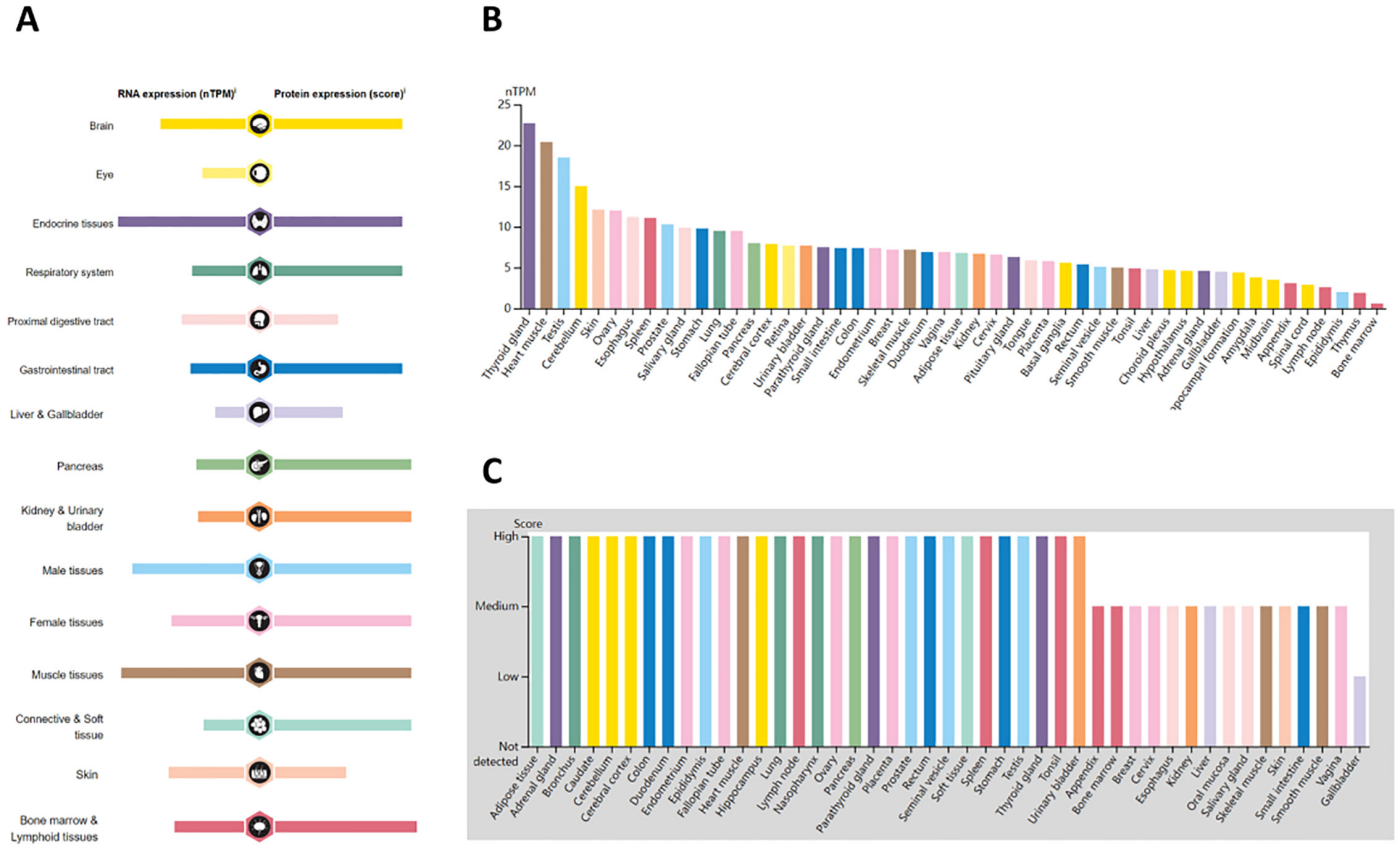
Overview of ASPP1 expression in human tissues and organs. **(A)** Expression of ASPP1 mRNA and protein in human tissues and organs; **(B)** A summary of ASPP1 mRNA expression by nTPM in different parts of the body; **(C)** Summary of ASPP1 protein expression at different levels of expression in different human tissues.

**Figure 2:**
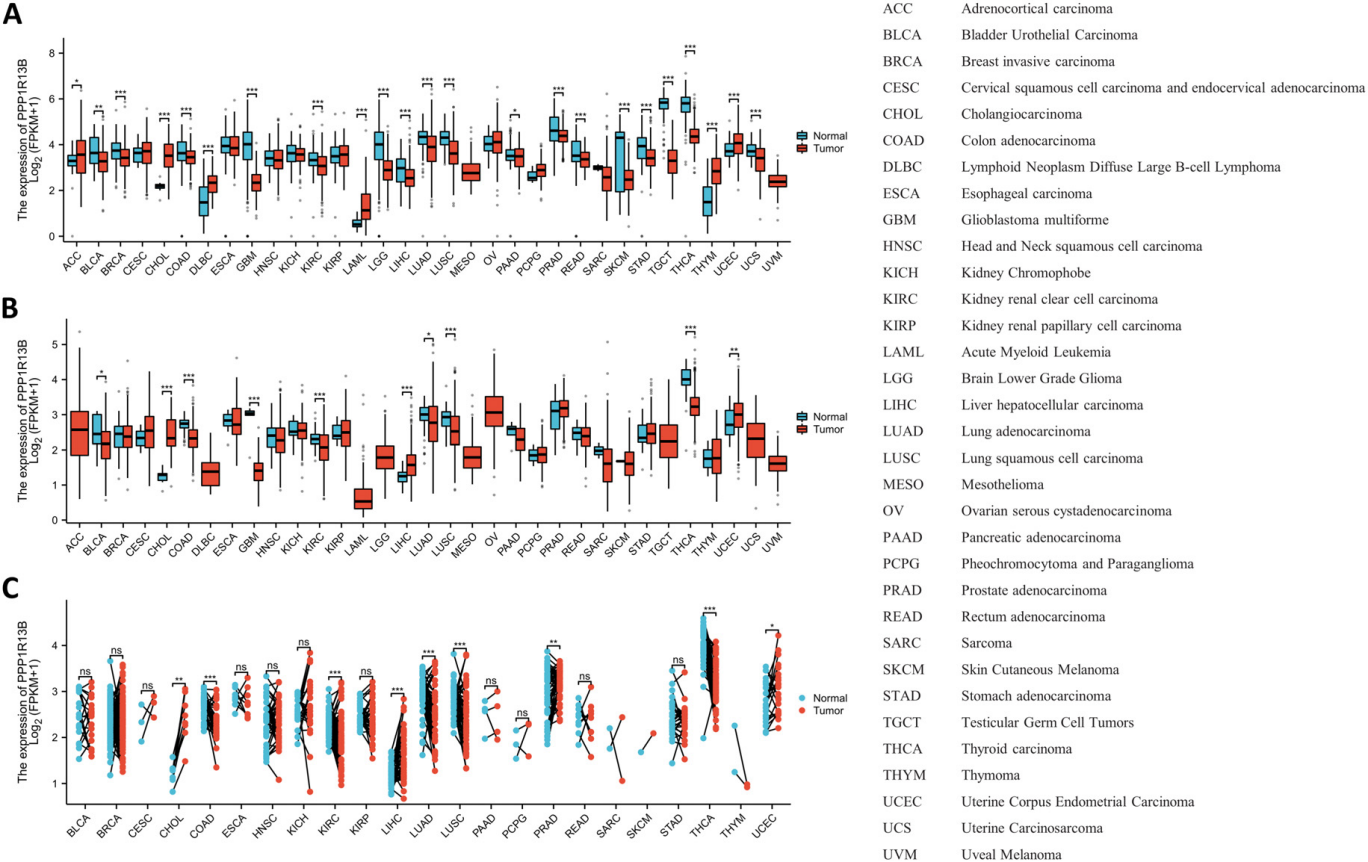
An analysis of ASPP1 gene expression in pan-cancer. **(A)** Analysis of unpaired samples for ASPP1 expression in 33 cancers and 33 normal tissues; **(B)** Unpaired sample analysis of the 33 cancers and paracancerous tissues shows differential expression of ASPP1; **(C)** An analysis of 18 cancers and 18 paracancerous tissues was performed for the ASPP1 mRNA expression. ^∗^p < 0.05, ^∗∗^p < 0.01, ^∗∗∗^p < 0.001. ns, not significant.

The expression landscape of ASPP1 in humans is shown in **Figure 1A**. ASPP1 mRNA and protein are widely expressed in a variety of tissues and organs. A majority of the mRNA from the ASPP1 gene is expressed in thyroid glands, heart muscle, testes, cerebellum, skin, ovary, and esophageal, spleen, prostate and salivary glands. (**Figure 1B**). High score tissues with ASPP1 protein level expression include adipose tissue, adrenal glands, bronchi, caudates, cerebellums, cortexes, colons, duodenums, endometriums, epididymis, and canaliculi. (**Figure 1C**). Analysis of unpaired samples from 33 cancer and normal tissues revealed that ASPP1 expression was significantly reduced in most cancer types, including COAD, GBM, BLCA, BRCA, LUSC, PAAD, READ, KIRC, LGG, LIHC, LUAD, TGCT, THCA, SKCM, STAD and UCS. Conversely, increased ASPP1 mRNA level expression was observed in DLBC, LAML, ACC, CHOL, THYM, and UCEC. No significant differences were detected in KIRP, MESO, CESE, ESCA, HNSC, KICH, OV, PCPG, SARC, or UVM. In comparison with paracancerous tissue, ASPP1 mRNA expression was significantly lower in BLCA, COAD, GBM, KIRC, LUAD, LUSC, THCA and significantly higher in CHOL, LIHC, UCEC. The expression of ASPP1 between cancers and normal tissues in paired sample has increased significantly Lower in COAD, KIRC, LUAD, LUSC, THCA and significantly higher in CHOL, LIHC, PRAD and UCEC. There was no significant difference shown in BLCA, BRCA, KICH, KIRP, PAAD, PCPG, CESC, ESCA, HNSC, READ, SARC, SKCM, STAD, THYM (p > 0.05) (**Figure 2**).

### 3.2 Exploring the diagnostic potential of ASPP1 in various cancer types

**Figure 3:**
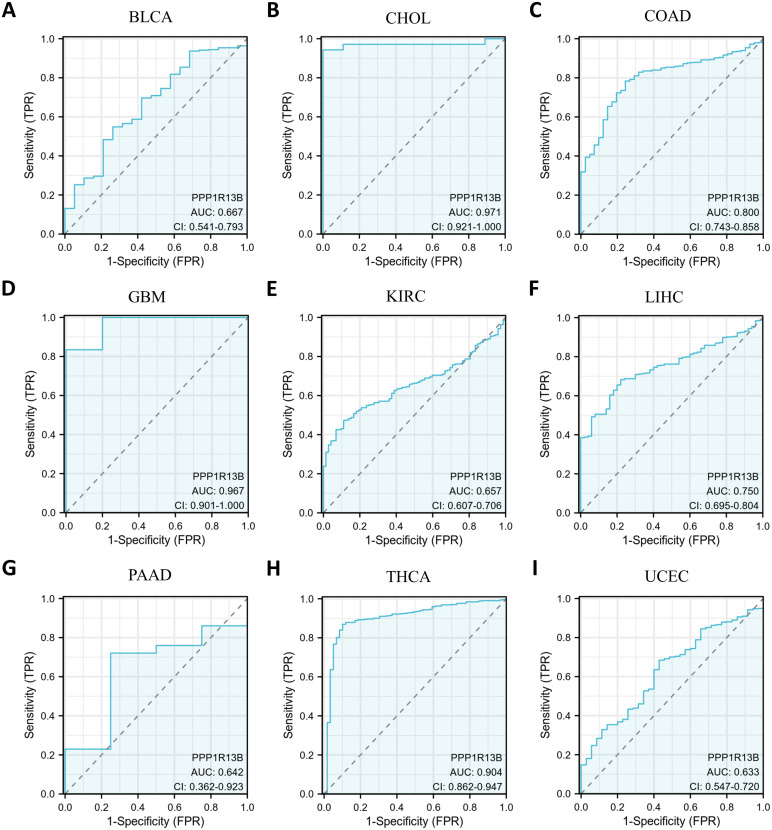
Curve of Receiver Operator Characteristics (ROC) for ASPP1 in 9 cancers. For ASPP1, cancers with an AUC > 0.6 are considered. **(A)** BLCA. **(B)** CHOL. **(C)** COAD. **(D)** GBM. **(E)** KIRC. **(F)** LIHC. **(G)** PAAD. **(H)** THCA. **(I)** UCEC.

As is the Diagnostic Value shown in **Figure 3**, COAD (AUC=0.8) and LIHC (AUC=0.75) with an AUC exceeded 0.7 were considered moderately diagnostic, and those with an AUC exceeded 0.9 were regarded as highly diagnostic contain CHOL (AUC=0.971), GBM (AUC=0.967), THCA (AUC=0.904) which displayed high diagnostic value.

### 3.3 Survival analysis of ASPP1 in cancers

**Figure 4:**
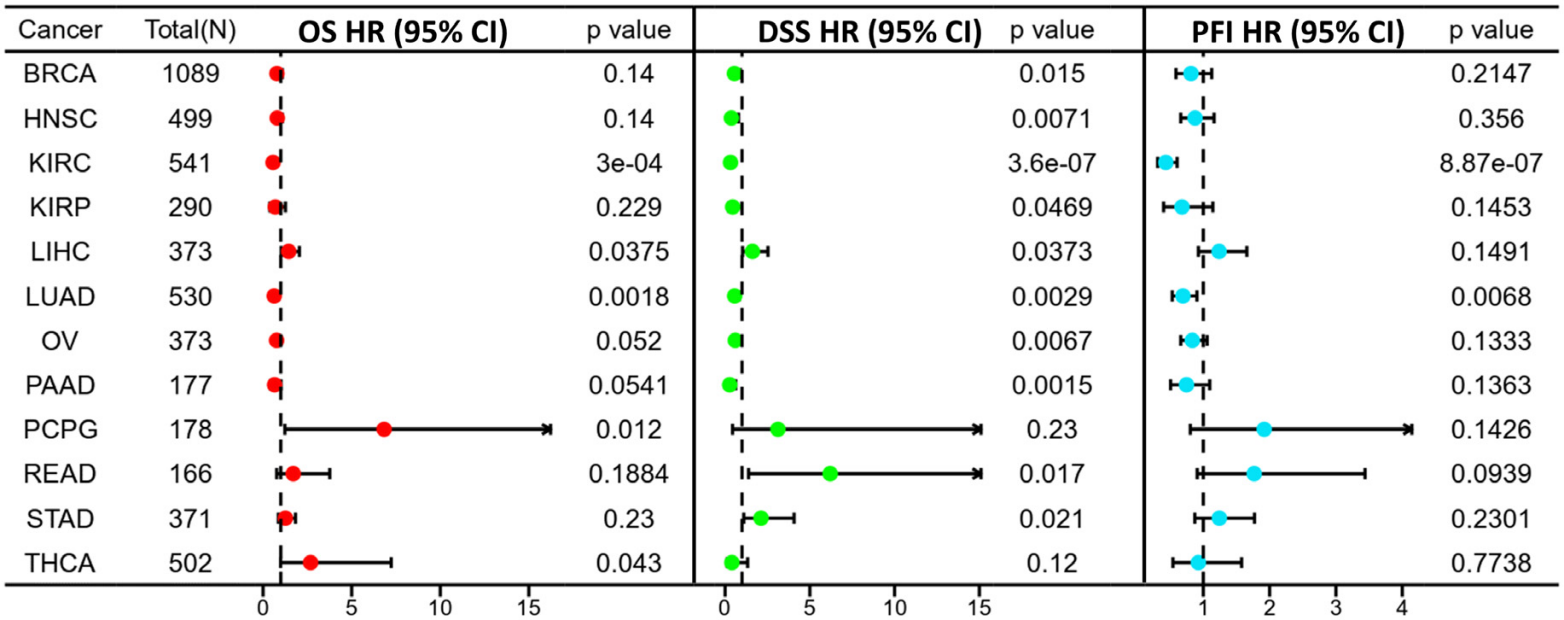
A forest plot illustrating ASPP1 OS (red), DSS (green), and PFI (blue) in 12 cancers. using the K-M method.

**Figure 5:**
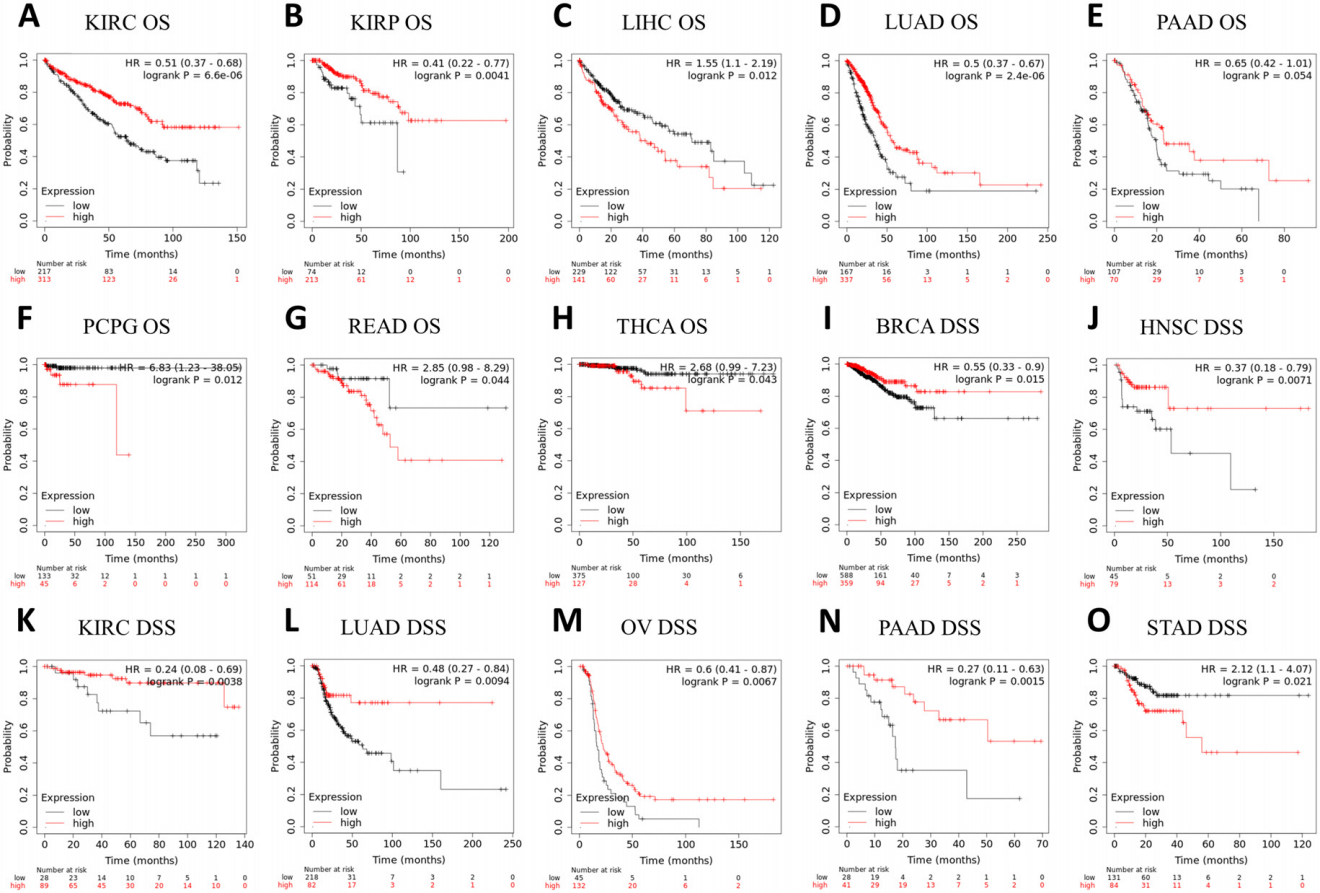
A correlation between ASPP1 and prognosis in 15 cancers. **(A-H)** The OS K-M curve for ASPP1 in 8 cancer types. **(I-O)** An DSS analysis of ASPP1 K-M curves in 7 cancers. The X-axis unit is month.

Prognosis can be predicted using the Kaplan–Meier survival analysis and display meaningful values using forest plots. ASPP1 expression was associated significantly with OS in 8 cancers using Cox regression analysis with OS, DSS, and PFI analysis of cancers. In KIRC, KIRP, LUAD, PAAD, high ASPP1 expression patients group overall survival rates and BRCA, HNSC, KIRC, LUAD, OV, PAAD DSS survival rates were statistically better than low expression group. The LIHC, PCPG, READ, THCA OS survival rates and STAD DSS survival rates were worse in cancers (**Figures 4**, **5**).

### 3.4 Expression of ASPP1 varies with cancer stage, immune status and molecular subtype in 33 cancers

**Figure 6:**
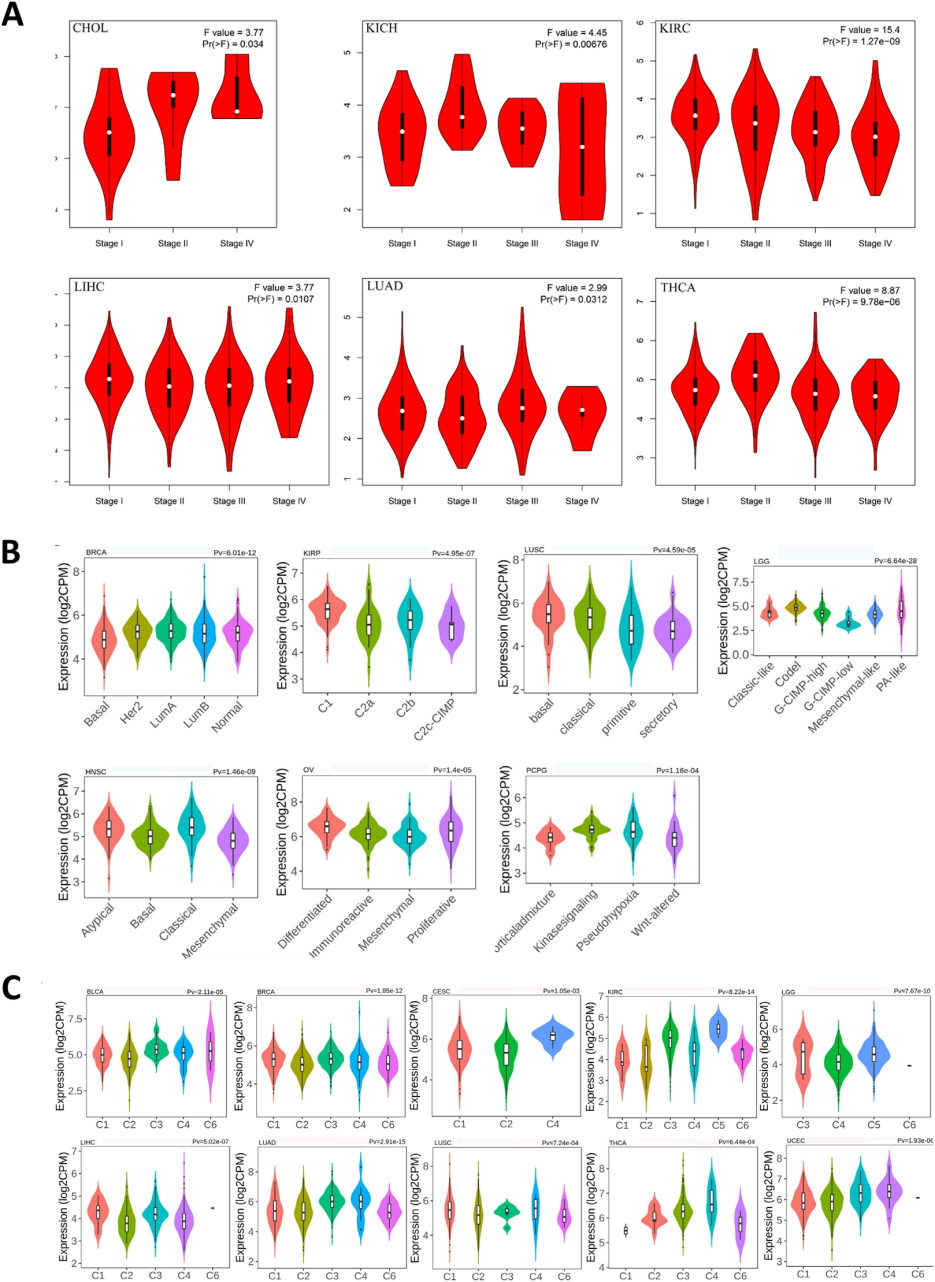
Correlations between ASPP1 expression and cancer stage, molecular subtypes, immune subtypes in cancers. **(A)**CHOL, KICH, KIRC, LIHC, LUAD, THCA stages plots. **(B)** Molecular subtypes in BRCA, KIRP, LUSC, LGG, HNSC, OV, PCPG. **(C)** Immune subtypes in LGG, LIHC, BLCA, BRCA, CESC, KIRC, LUAD, LUSC, THCA, UECE.

Towards to a better understanding of ASPP1 in cancers, we analyzed the ASPP1 expression in different stages, immune subtype and molecular subtype. The results show that ASPP1 expresses differs significantly differently in 6 cancer stages about CHOL (stage I, II, IV), KICH (stage I, II, III, IV), KIRC (stage I, II, III, IV), LIHC (stage I, II, III, IV), LUAD(stage I, II, III, IV), THCA(stage I, II, III, IV) and significantly differently in 7 cancer molecular subtypes include BRCA (Basal, Her 2, Lumu A, Lumu B, Normal), KIRP (C1, C2a, C2b, C2c-CIMP), LUSC (Basal, Classical, Primitive, Secretory), LGG (Classic-like, Codel, G-CIMP-high, G-CIMP-low, Mesenchymal-like, PA-like), HNSC (Atypical, Basal, Classical, Mesenchymal), OV (Differentiated, Immunoreactive, Mesenchymal, Proliferative), PCPG (Corticaladmixture, Kinasesignaling, Pseudohypoxia, Wnt-altered) (**Figures 6A, B**). For immune subtypes, ASPP1 expresses significantly differently in 10 cancer types, including BLCA (5 subtypes), BRCA (5 subtypes), CESC (3 subtypes), KIRC (6 subtypes), LGG (4 subtypes), LIHC (5 subtypes), LUAD (5 subtypes), LUSC (5 subtypes), THCA (5 subtypes), UCEC (5 subtypes) (**Figure 6C**).

### 3.5 Genetic alteration of ASPP1

**Figure 7:**
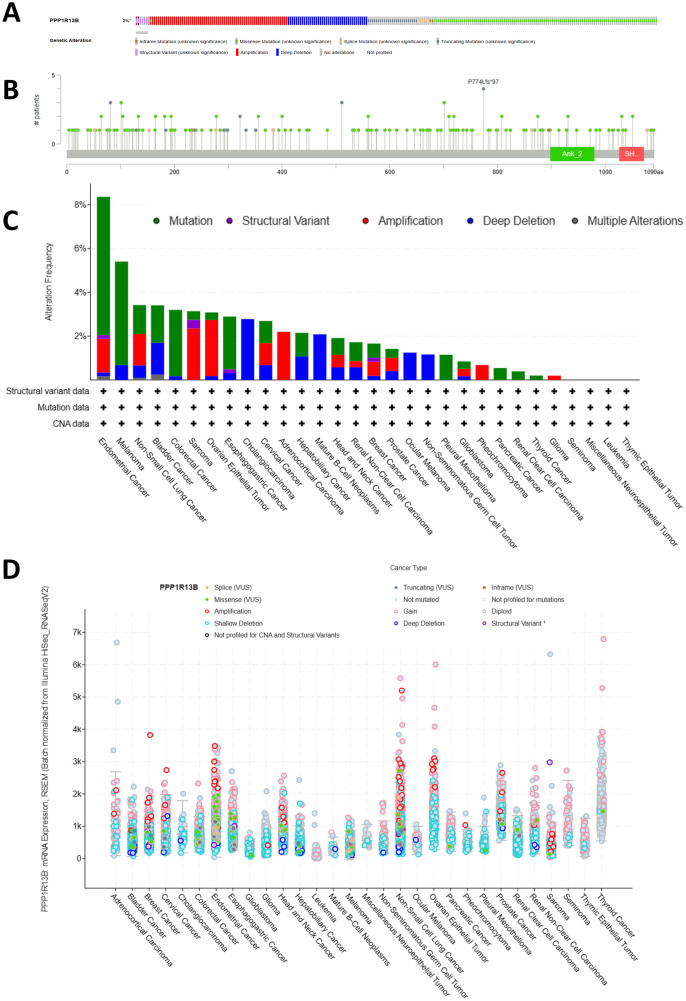
Genetic alterations of ASPP1 in pan-cancer. **(A)** The frequency of gene alterations in ASPP1. **(B)** An overview of gene alterations in ASPP1. **(C)** The frequency of mutations in ASPP1 in cancers has changed. **(D)** An analysis of the number and type of mutations in the ASPP1 gene in cancers.

A genetic investigation of ASPP1 expression across various cancers was performed using 10,967 samples from 32 studies in the TCGA Pan-Cancer Atlas via cBioPortal. Diverse genetic mutations were identified, each potentially contributing to cancer initiation and progression. Amplification, deep deletion, missense mutations, and truncating mutations were the most prevalent genetic alterations observed in ASPP1 as shown in **Figure 7A**. Mutations accounted for the majority of all altered types, including mutations, structural variants, amplifications, and deep deletions. As shown in **Figure 7B**, 177 mutations were found involving ASPP1 in pan cancers, including 136 missense mutations, 25 truncating mutations, 7 Splice mutations, 7 Fusion mutations and 2 Inframe mutations. The ASPP1 mutation were most commonly seen in UCEC, SKCM, BLCA, COAD, ESCA. Amplification was most commonly seen in UCEC, NSCLC, SARC, OV and ACC. As shown in **Figure 7C**, deep deletions are most commonly seen in BLCA, CHOL, and mature B cell neoplasms. A shallow deletion, an amplification, and a gain in ASPP1 mRNA expression was common among nearly all 30 cancers (**Figure 7D**).

### 3.6 PPI, functional and gene set enrichment of ASPP1 in cancers

**Figure 8:**
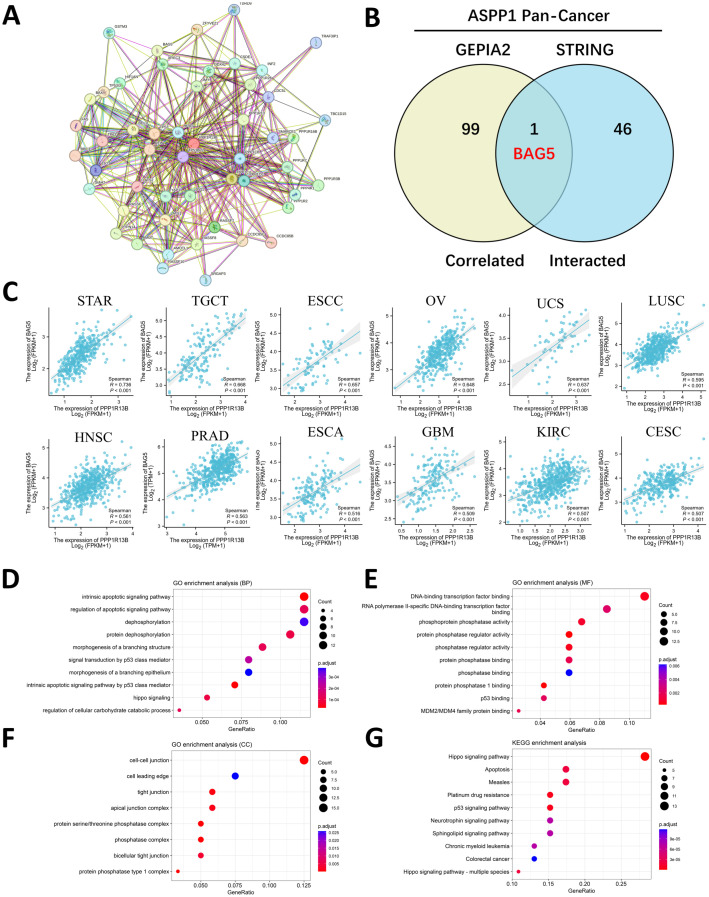
An enrichment analysis of genes related to ASPP1. **(A)** An analysis of the ASPP1 interaction network using the STRING database. **(B)** A correlation analysis between ASPP1-correlated genes and ASPP1-interacted genes. **(C)** An analysis of the correlation between ASPP1 and BAG5 in various cancers. **(D-G)** ASPP1 and interacted genes enriched for GO/KEGG pathways.

The STRING website was searched at the given threshold to compile an interactive map of PPI interaction based on 47 genes closely linked to ASPP1 (**Figure 8A**). The 47 interacting genes from STRING were compared to the top 100 related genes from GEPIA2 of ASPP1 using an interactive Venn diagram. In the repeat region, only BAG5 (BCL2-associated athanogene 5) was duplicated, indicating that ASPP1 plays an important role in cancer cell apoptosis as shown in **Figure 8B**. Next, we analyzed the correlation of molecular expression between ASPP1 and BAG5 in different tumors using spearman analysis. A strong correlation was found between ASPP1 and BAG5 in almost all cancer types except LAML (R=-0.034) indicating that ASPP1 and BAG5 have strong relation in cancers (**Figure 8C**). To determine the role of ASPP1 in cancer function, KEGG pathway analysis and GO enrichment analysis were conducted using ASPP1 correlated and interacted genes. In GO enrichment analysis (BP), regulation of apoptotic signaling pathways, dephosphorylation and intrinsic apoptotic signaling pathways were three top GO enrichment pathways. A number of DNA-binding transcription factors, RNA polymerase II-specific DNA-binding transcription factors, and phosphoprotein phosphatase activities had the highest GO enrichment analysis (MF). Cell-cell junction, cell leading edge, and tight junction were the top GO enrichment analysis (MF). As shown in the KEGG analysis, Hippo signaling pathways, apoptosis, and measles appeared to be the top three enriched pathways. ASPP1 is mainly associated with apoptosis, dephosphorylation, DNA-binding transcription, phosphoprotein activity, cell migration, and cell-cell junction functions based on the results of GO/KEGG enrichment analysis (**Figures 8D–G**).

### 3.7 Immune infiltration analysis in cancers

**Figure 9:**
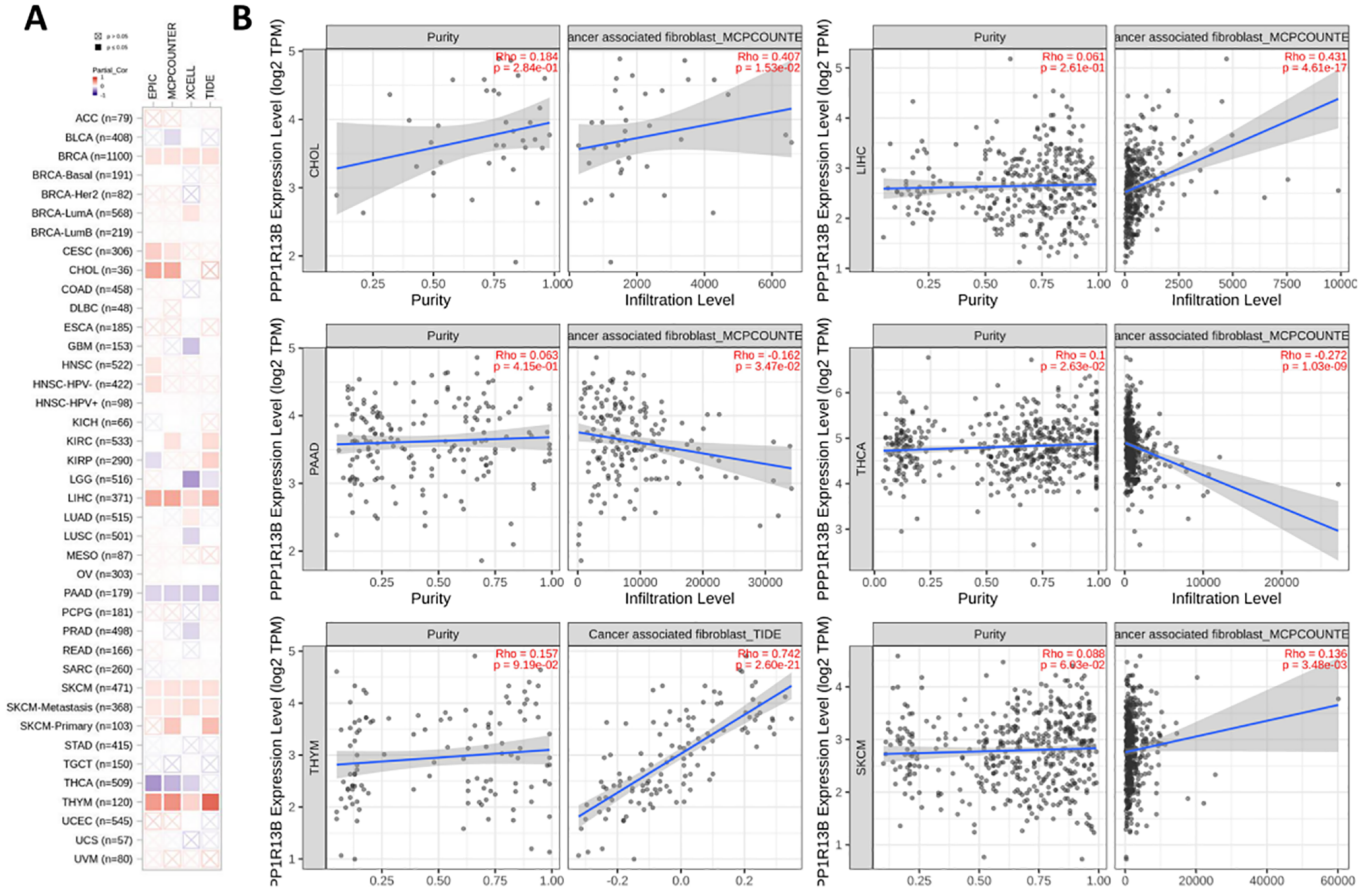
ASPP1 expression is associated with immune infiltration of cancer-associated fibroblasts. **(A)**. Correlation of ASPP1 expression with CAF (TIMER2.0). **(B)** Correlation of ASPP1 expression with purity and infiltration level in cancers.

The immune microenvironment plays an important role in tumor development which cancer associated fibroblasts could promote tumor progression. We observed that the ASPP1 expression has correlates positively with estimates value of cancer-associated fibroblast infiltration in CHOL, LIHC, THYM, SKCM and negative correlation with PAAD, THCA. A method of evaluating immune cell infiltration was TIDE algorithm shows the same results in CHOL, LIHC, THYM, SKCM, PAAD and THCA (**Figure 9**).

### 3.8 ASPP1 immunogenomics analysis in cancers

**Figure 10:**
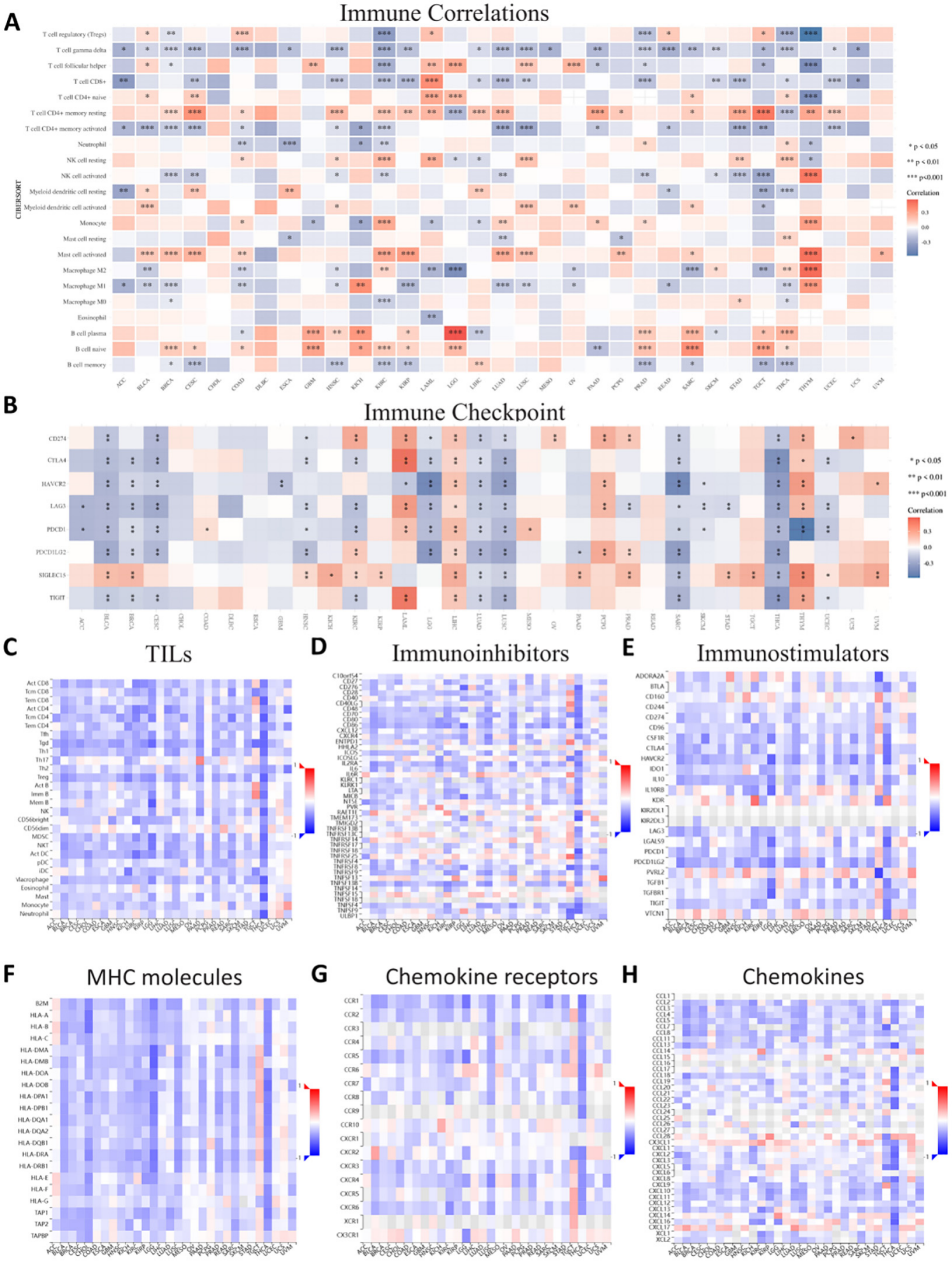
Correlations between ASPP1 and immune-related genes in cancer. **(A)** Immune Correlations. **(B)** Immune checkpoint. **(C)** TILs. **(D)** Immunoinhibitors. **(E)** Immunostimulators. **(F)** MHC molecules. **(G)** Chemokine receptors. **(H)** Chemokines. Red represents positive correlation, blue represents negative correlation, and the darker the color, the stronger the correlation. *p < 0.05, **p < 0.01, ***p < 0.001.

To further evaluate ASPP1’s relationship with immune regulation and immune infiltration, we built 7 different types of heat maps to compare ASPP1 to markers associated with immune cells. A positive correlation was observed between ASPP1 expression and the immune score in multiple tumor tissues, whereas a negative correlation was observed between ASPP1 and CD4+ T cells at rest, B cells at plasma, and B cells at native. In many cancer types, ASPP1 exhibits a negative correlation with activated CD4+ T cells, gamma delta T cells, CD8+ T cells, and memory B cells. To assess the relationship between ASPP1 and immune checkpoint-related genes, a heatmap was generated for several key genes. Results show that A positive correlation exists between ASPP1 expression and immune checkpoint genes in THYM, PCPG, LIHC, LAML, KIRC and negatively correlated with THCA, SARC, LUSC, LUAD, LGG, HNSC, CESC, BRCA, BLCA. A negative correlation was seen between ASPP1 and most TILs, immunoinhibitors and immunoostimulators especially in THCA. Immunostimulators KIR2DL1 and KIR2DL3 lack sufficient data in most cancers. According to ASPP1, MHC molecules are positively correlated with TGCT but negatively correlated with other cancers. For most chemokine receptors, ASPP1 expressed a negative correlation with KIRC, COAD, LGG, LUSC, THCA, and positively correlated with TGTC. There are a few chemokines that are positively correlated with ASPP1 in cancers, such as CCL28, CX3CL1, CXCL14, and CXCL17. It was found that ASPP1 was negatively correlated with chemokines in THCA (**Figures 10A–H**).

### 3.9 Vitro experiment to validate

**Figure 11:**
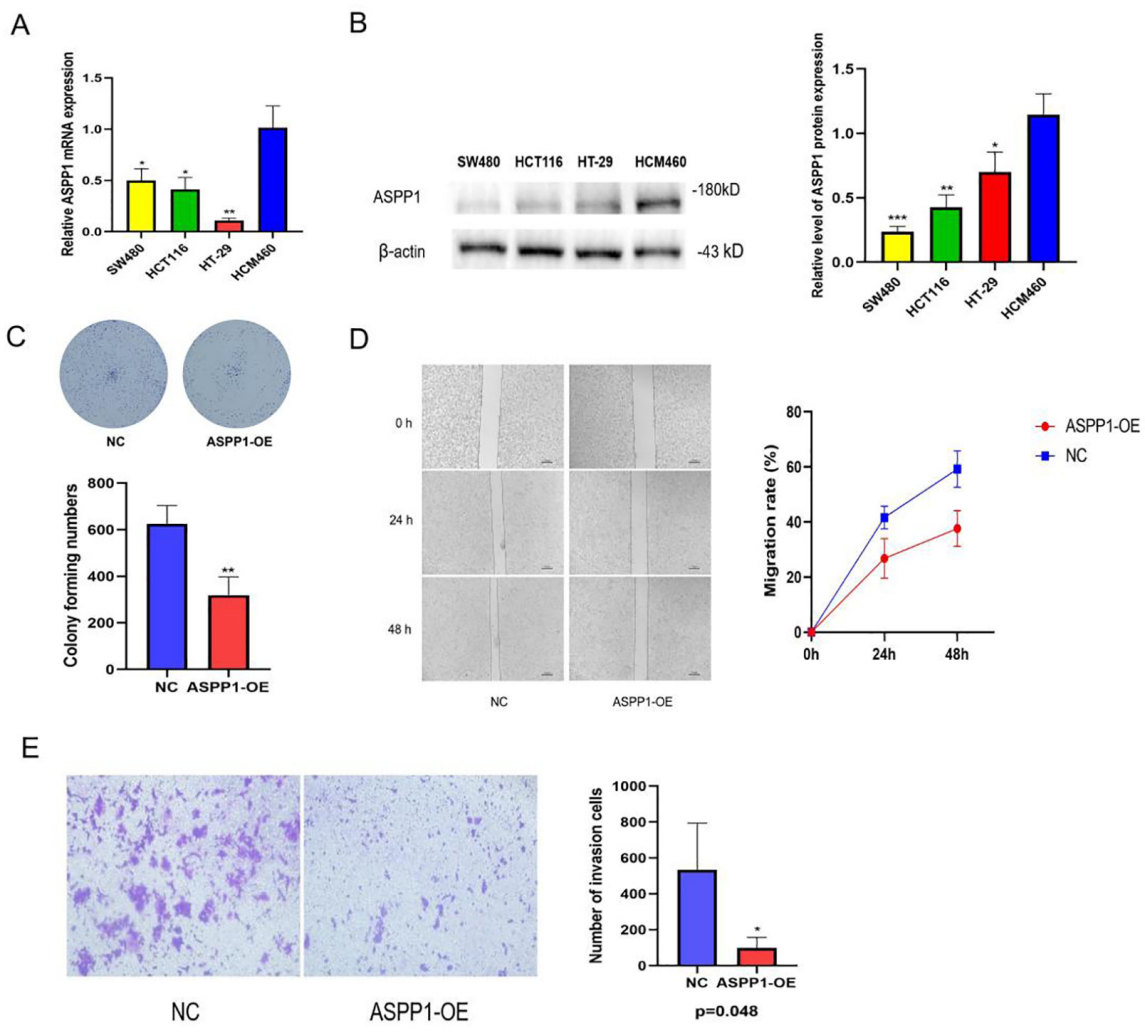
Expression and functional verification of ASPP1 in colorectal cancer cells. **(A)** Relative ASPP1 mRNA expression levels in different colorectal cancer cell lines. **(B)** Western blot analysis of ASPP1 protein expression in colorectal cancer cell lines. **(C)** Colony formation assay of NC and ASPP1-OE cells. **(D)** Wound healing assay showing migration capacity of NC and ASPP1-OE cells at 0, 24, and 48 hours. **(E)** Transwell invasion assay comparing the invasive potential of NC and ASPP1-OE cells. *p < 0.05; **p < 0.01. ***p < 0.001.

The relative mRNA expression levels of ASPP1 in various colorectal cancer cell lines (SW480, HCT116, HT-29, HCM460) were assessed using quantitative RT-PCR. The results indicate that ASPP1 expression is significantly lower in SW480, HCT116, and HT-29 cells compared to HCM460 cells (**Figure 11A**). Analyses of WB further confirmed the differential expression of ASPP1 protein among these cell lines, revealing that SW480, HCT116, and HT-29 exhibit reduced ASPP1 protein levels relative to HCM460, with statistical significance (p < 0.05, **Figure 11B**). Colony formation assays demonstrated a notable decrease in colony numbers in ASPP1 overexpression (ASPP1-OE) cells compared to the negative control (NC) cells (**Figure 11C**). Additionally, wound healing assays tracked the migration process over time, showing a significant reduction in the migration rate of ASPP1-OE cells relative to NC cells (**Figure 11D**). Finally, crystal violet staining of invasive cells revealed that the ASPP1-OE group had significantly fewer invasive cells than the NC group (p < 0.05, **Figure 11E**).

## 4 Discussion

Apoptosis-stimulating protein of p53, also known as ASPP1 (PPP1R13B), plays an important role in regulating apoptosis and has been linked in multiple cancer types (29). It promotes the release of pro-apoptotic proteins from the promoter of pro-apoptotic proteins, enhances the DNA-binding and transactivation functions of P53, and promotes the expression of downstream pro-apoptotic genes (BAX, PUMA) (9). There has been a significant downregulation in the expression level of ASPP1 in various forms of human cancer, including acute lymphoblastic leukemia, breast cancer, hepatocellular carcinoma bearing hepatitis B virus, clear cell renal cell carcinoma, and colorectal cancer (CRC) (10, 11, 15–17). Downregulation of ASPP1 may enable NF-κB to promote invasion and migration by activating Snail2, ultimately leading to epithelial-mesenchymal transition (EMT) (30). Here, we performed a thorough analysis of the ASPP1 gene across multiple cancer types. Our aim was to explore the potential role of ASPP1 in cancer and its implications for clinical practice.

According to our findings, ASPP1 expression is dysregulated in several types of cancer. This study demonstrated significant downregulation of ASPP1 in GBM, KIRC, LGG, BLCA, COAD, LIHC, BRCA, PAAD, SKCM, STAD, LUAD, PRAD, READ, LUSC, UCS, THCA and TGCT, suggesting this protein might be involved in tumor suppression. On the other hand, ASPP1 has been found to be upregulated in certain cancer types, such as ACC, CHOL, DLBC, LAML, UCEC and THYM. Based on these findings, ASPP1 may assume a context-dependent role in the progression of various cancer types. A further examination of ASPP1 expression and clinicopathological characteristics of cancer patients was conducted. In light of our findings, the AUC of ROC for five cancer types, including COAD, LIHC, CHOL, GBM and THCA, exceeded 0.7, indicating the high diagnostic effectiveness of ASPP1. A survival analysis showed that ASPP1 expression is correlated with DSS, OS and PFI in a number of tumor types. A lower ASPP1 expression in most cancers was associated with an adverse prognosis. As a result, to identify patients at high risk of metastasis and poor outcomes, ASPP1 downregulation may be a prognostic marker for cancer progression.

Mutations in ASPP1 are most commonly found in UCEC, SKCM, BLCA, COAD, and ESCA, suggesting that ASPP1 mutations might play oncogenic roles. Our functional enrichment analysis of ASPP1-associated genes provided insights into the molecular mechanisms underpinning ASPP1’s role in cancer. In our analysis, we found that ASPP1 regulates the cell cycle, repairs DNA, and initiates apoptosis among other biological processes. By modulating these key cellular processes, ASPP1 may exert its tumor-suppressive properties. Among the ASPP1-associated genes, we found significant enrichment of several signaling pathways, notably the Hippo and p53 pathways. Several of these pathways have been established as contributing to cancer development, supporting the hypothesis that ASPP1 is also involved in cancer development. Recent research has indicated that ASPP1 is significantly downregulated in colorectal cancer (CRC) cells, which influences p53-mediated apoptotic pathways and facilitates cancer progression (31). In alignment with these findings, as illustrated in Figures A and B, ASPP1 expression levels in colorectal cancer cell lines such as SW480, HCT116, and HT-29 were markedly lower compared to those in normal intestinal epithelial cells like HCM460, highlighting the potential role of ASPP1 in colorectal carcinogenesis.

Colony formation assays revealed that ASPP1 over express in CRC cells resulted in a substantial reduction in colony numbers, indicating its role as a growth inhibitor. Additionally, wound healing assays demonstrated decreased migration in ASPP1-overexpressing cells, reinforcing the notion that ASPP1 may play a critical role in curbing metastasis. These results suggest that the downregulation of ASPP1 enhances the proliferation, migration, and invasion of CRC cells, thereby contributing to cancer progression (11). Moreover, the reduction of cancer cell proliferation and migration associated with ASPP1 overexpression point to its potential as a therapeutic target in CRC.

Further investigation is warranted to clarify the underlying molecular processes by which ASPP1 operates in CRC, particularly its interactions with non-p53 pathways. Our findings support the assertion that ASPP1 functions as a tumor suppressor in CRC cells, with its reduced expression correlating with increased proliferation, migration, and invasion. Targeting ASPP1 may provide novel therapeutic strategies for patients with CRC.

## 5 Conclusions

In this study, we present a detailed investigation into the role of ASPP1 in cancer, confirming its expression and function in colorectal cancer cell lines. Dysregulation of ASPP1 has been documented across various cancer types and correlates with clinical characteristics and patient prognoses. Furthermore, our functional enrichment analysis highlights the significant involvement of ASPP1 in multiple signaling pathways and cellular processes that contribute to cancer progression. Given these findings, ASPP1 emerges as a promising prognostic marker and therapeutic target, particularly in colorectal cancer. Targeting ASPP1 may hold significant therapeutic potential in cancer treatment. Nonetheless, Additional study is required to clarify the specific mechanisms through which ASPP1 influences colorectal cancer cells.

## Data Availability

The raw data supporting the conclusions of this article will be made available by the authors, without undue reservation.
